# Supplementary material for: Causal Relationship between Aging and Anorexia Nervosa: A White-Matter-Microstructure-Mediated Mendelian Randomization Analysis
Source: Biomedicines. 2024 Aug 16;12(8):1874. doi: 10.3390/biomedicines12081874 (PMC11351342; doi:10.3390/biomedicines12081874)
Supplement: Supplementary file 1 [file biomedicines-12-01874-s001.zip › Supplementary Figure/Figure S4/Singe SNP plot of MR analysis for TL to ubm-b-2095.pdf]

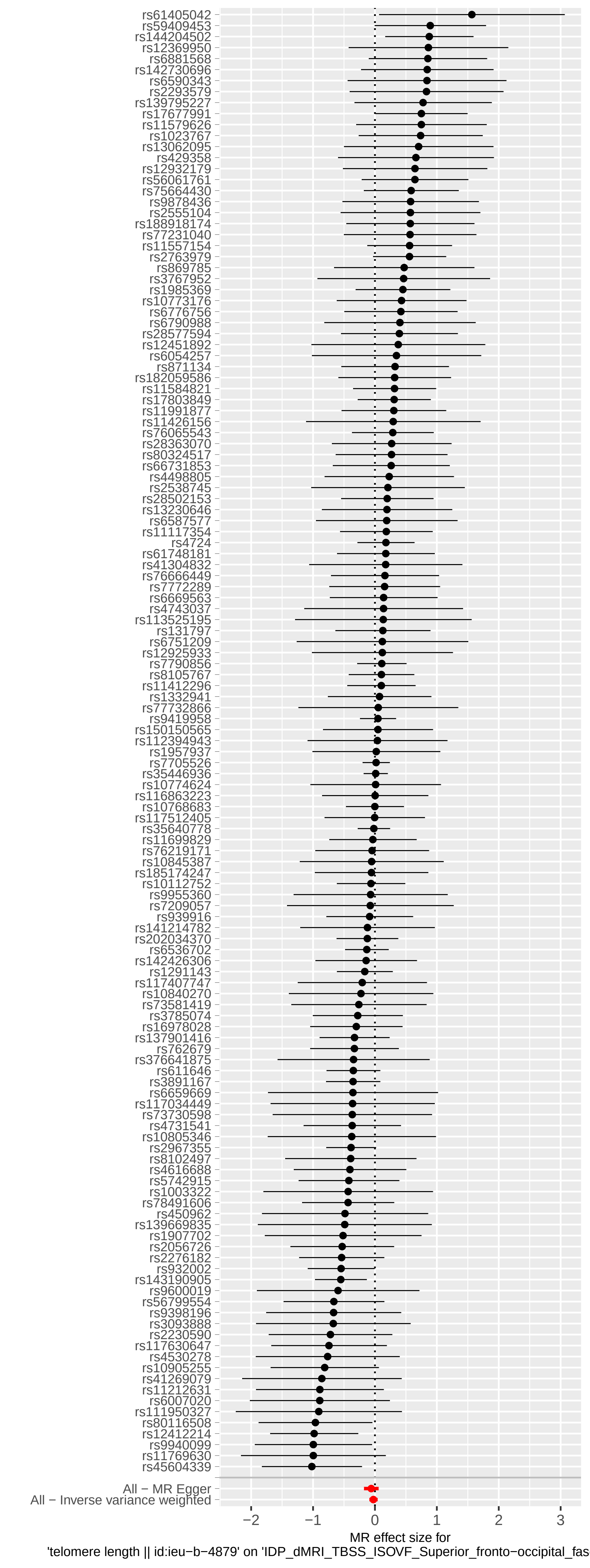

rs61405042
rs59409453
rs144204502
rs12369950
rs6881568
rs142730696
rs6590343
rs2293579
rs139795227
rs17677991
rs11579626
rs1023767
rs13062095
rs429358
rs12932179
rs56061761
rs75664430
rs9878436
rs2555104
rs188918174
rs77231040
rs11557154
rs2763979
rs869785
rs3767952
rs1985369
rs10773176
rs6776756
rs6790988
rs28577594
rs12451892
rs6054257
rs871134
rs182059586
rs11584821
rs17803849
rs11991877
rs11426156
rs76065543
rs28363070
rs80324517
rs66731853
rs4498805
rs2538745
rs28502153
rs13230646
rs6587577
rs11117354
rs4724
rs61748181
rs41304832
rs76666449
rs7772289
rs6669563
rs4743037
rs113525195
rs131797
rs6751209
rs12925933
rs7790856
rs8105767
rs11412296
rs1332941
rs77732866
rs9419958
rs150150565
rs112394943
rs1957937
rs7705526
rs35446936
rs10774624
rs116863223
rs10768683
rs117512405
rs35640778
rs11699829
rs76219171
rs10845387
rs185174247
rs10112752
rs9955360
rs7209057
rs939916
rs141214782
rs202034370
rs6536702
rs142426306
rs1291143
rs117407747
rs10840270
rs73581419
rs3785074
rs16978028
rs137901416
rs762679
rs376641875
rs611646
rs3891167
rs6659669
rs117034449
rs73730598
rs4731541
rs10805346
rs2967355
rs8102497
rs4616688
rs5742915
rs1003322
rs78491606
rs450962
rs139669835
rs1907702
rs2056726
rs2276182
rs932002
rs143190905
rs9600019
rs56799554
rs9398196
rs3093888
rs2230590
rs117630647
rs4530278
rs10905255
rs41269079
rs11212631
rs6007020
rs111950327
rs80116508
rs12412214
rs9940099
rs11769630
rs45604339
All − MR Egger
All − Inverse variance weighted
−2
−1
0
1
2
3
MR effect size for
'telomere length || id:ieu−b−4879' on 'IDP_dMRI_TBSS_ISOVF_Superior_fronto−occipital_fas
